# Evaluation of sodium nitroprusside for controlled hypotension in children during surgery

**DOI:** 10.3389/fphar.2015.00136

**Published:** 2015-07-06

**Authors:** David R. Drover, Gregory B. Hammer, Jeffrey S. Barrett, Carol A. Cohane, Tammy Reece, Anne Zajicek, Scott R. Schulman

**Affiliations:** ^1^Department of Anesthesia, Perioperative and Pain Medicine, Stanford University School of MedicineStanford, CA, USA; ^2^Clinical Pharmacology and Therapeutics Division, The Children's Hospital of Philadelphia and Department of Pediatrics, University of Pennsylvania Medical SchoolPhiladelphia, PA, USA; ^3^Duke Clinical Research Institute, Duke University Medical CenterDurham, NC, USA; ^4^The Eunice Kennedy Shriver National Institute of Child Health and Human Development, National Institutes of HealthBethesda, MD, USA; ^5^Department of Anesthesia and Perioperative Care, University of California, San FranciscoSan Francisco, CA, USA

**Keywords:** nitroprusside, pediatric, BPCA, cyanide toxicity, controlled hypotension

## Abstract

**Purpose:** (1) To define the onset and offset of the blood-pressure-lowering effects of sodium nitroprusside (SNP) for use in developing instructions for dose titration in children undergoing a surgical or medical procedure, and (2) to assess the safety of SNP administration in pediatric patients requiring controlled reduction of blood pressure.

**Methods:** We conducted a randomized, double-blind, parallel-group, dose-ranging, effect-controlled, multicenter study of intravenous (IV) infusions of SNP in pediatric patients <17 years, who required controlled hypotension for at least 2 h while undergoing a surgical or medical procedure. A blinded SNP dose of 0.3, 1, 2, or 3 μg/kg/min was infused for 30 min, followed by open-label administration for at least 90 min. Both infusions were titrated to effect.

**Results:** The final intent-to-treat group comprised 203 patients. Significant reductions in mean arterial pressure (MAP) from baseline were observed for all four doses at 20 and 25 min after the start of infusion (*p* ≤ 0.009 and *p* ≤ 0.010 for each time, respectively). Overall, 98.5% of the patients achieved the target MAP; 72.9% first achieved the target MAP during the blinded infusion. The mean infusion rate at target MAP was 1.07 μg/kg/min.

**Conclusion:** We determined that 0.3 μg/kg/m is a reasonable starting dose for SNP in pediatric patients requiring controlled hypotension. The infusion rate can then be increased to achieve the desired reduction in blood pressure. On the basis of our results, we found an average infusion rate of 1 μg/kg/min might be appropriate. Of note, no cyanide toxicity was reported, and no measureable cyanide levels were detected in any blood samples obtained during the study. http://clinicaltrials.gov/show/NCT00135668.

## Introduction

Sodium nitroprusside (SNP) was first discovered in 1850, but its hypotensive effects were not noticed until 1929. Page et al. reported its first therapeutic use in 1955 (Page et al., 1955), and in 1962 Moraca et al. were the first to use SNP clinically to induce deliberate hypotension during surgery (Moraca et al., 1962). Since then, SNP has been widely used to control blood pressure in infants and children in the perioperative period, despite the lack of FDA approval for this age group in the United States. Sodium nitroprusside is the first medication to gain regulatory approval through the Best Pharmaceuticals in Children's Act (BPCA), which was enacted in 2007.

Two million surgical procedures are performed annually on children <15 years of age, and general anesthesia is administered in the vast majority of cases. Controlled hypotension is used in a select group of patients undergoing certain orthopedic, neurosurgical, craniofacial, ear-nose-and-throat, and burn procedures. Controlled hypotension is the stepwise, deliberate reduction of systemic arterial blood pressure in order to decrease blood loss and the need for blood transfusion and to improve visibility in the surgical field.

Blood pressure control in children is also a concern in the intensive care unit (ICU), where it is often necessary to manage arterial pressure during periods of acute physiologic stress, which occurs during and after certain surgical and medical procedures, such as aortic coarctation repair, pulmonary valve autograft (Ross) procedures, and solid organ transplantation. Control of systemic arterial pressure is also indicated in cases of hypertension associated with renal disease, drug therapy (corticosteroids and immunosuppressive agents), and extracorporeal membrane oxygenation (ECMO).

A wide variety of drugs of various therapeutic classes have been used to induce controlled hypotension in the operating room and to prevent or treat hypertension in the pediatric ICU. These drug classes include calcium channel blockers (Tobias et al., 1996), beta-adrenergic antagonists (Kay et al., 2001), ganglionic blockers (Du Toit, 1970; Gallagher and Milliken, 1979), inhalation anesthetics (Tobias, 1998), and direct-acting vasodilators such as nitroglycerin and SNP (Jones and Cole, 1968; Taylor et al., 1970; Wilson et al., 1971; Tinker and Michenfelder, 1976; Kaplan et al., 1980; Groshong, 1996; Sinaiko, 1996). Despite the frequency of their use, vasodilating agents have not generally been systematically studied in children. Sodium nitroprusside is a direct-acting vasodilator commonly used for blood pressure control. It induces vascular smooth muscle relaxation via nitric oxide production. Because SNP metabolism also liberates cyanide (CN), CN toxicity is a concern when SNP is infused at high doses and/or for prolonged periods of time, but there are little data to inform clinicians of the risks of specific infusion regimens across pediatric age groups.

We performed a randomized, double-blind, parallel-group, dose-ranging, effect-controlled, multicenter study examining the effects of SNP in pediatric patients requiring controlled hypotension for at least 2 h during a surgical or medical procedure. Our aim was to establish the starting and maximum infusion rates that afford optimum blood pressure control and to develop a safe dosing regimen in children. The specific objectives were to (1) describe the relationship between the infusion rate of SNP and changes in blood pressure (2) to define the onset and offset of blood-pressure-lowering effects of SNP to be used to provide adequate instructions for dose titration in the pediatric population undergoing a surgical or medical procedure, and (3) to assess the safety of SNP administration in pediatric patients requiring controlled reduction of blood pressure.

## Materials and methods

The study was approved by the IRB at each of the 11 study research centers before enrollment. Parental informed consent and subject assent, when applicable, were obtained for all participants. Subjects were male and female patients, from birth through 16 years, who required intra-operative deliberate hypotension for at least 2 h. Enrollment was stratified into 5 age groups: neonates (<30 days), infants and toddlers (30 days to <2 years), pre-school children (2 to <6 years), school-age children (6 years of age to <Tanner Stage III), and adolescents (Tanner Stage III through 16 years). Complete inclusion and exclusion criteria are detailed in Table 1.

**Table 1:** **Inclusion and exclusion criteria**.

**S. No**.	**Inclusion criteria**	**Exclusion criteria**
1	Patient was < 17 years of age	Patient had a known allergy to SNP
2	Neonates must have been full-term gestation and have had a body weight of at least 2.5 kg	Patient had a known mitochondrial cytopathy with a disorder of oxidative phosphorylation or of respiratory chain enzymes
3	Patient required pharmacologically-induced hypotension for acute blood pressure management for surgery or other invasive procedure, e.g., cerebral artery embolization	Patient had a contraindication to vasodilator therapy for control of blood pressure during surgery or procedures
4	Duration of the patient's controlled hypotension was expected to be ≥ 2 h	Patient had participated in other clinical trials for investigational drugs and/or devices within 30 days prior to enrollment
5	Patient required general anesthesia with endotracheal intubation or IV sedation without endotracheal intubation	Patient had any serious medical condition which, in the investigator's opinion, was likely to interfere with study procedures
6	Patient required placement of intra-arterial line during the surgical or medical procedure	Patient was moribund (death likely to occur within 48 h)
7	The patient's parent or legal guardian gave permission (informed consent) and patient gave assent when appropriate	Patient had a positive result for the urine or serum human chorionic gonadotropin (HCG) test administered at screening

The following clinical parameters were carefully outlined prospectively before beginning patient enrollment: (a) Controlled hypotension was defined as a reduction in mean arterial blood pressure (MAP) to a target value specified by the investigator prior to the start of SNP infusion and in keeping with the clinical presentation and needs of the patient (b) the screening MAP was defined as the calculated MAP from the blood pressure recorded at the preoperative visit taken within 14 days of the planned operative or other procedure where hypotension was required (c) the baseline MAP was defined as the blood pressure recorded just before the blinded study drug administration period following at least a 5-min period of stable anesthesia (no changes in dosages of anesthetic agents) and after the completing the initial fluid rehydration infusions, and (d) the minimum target MAP value was 50 mmHg for patients ≥30 days of age and 40 mmHg for patients <30 days of age.

Previous treatment effects on blood pressure in a pediatric population suggested that a reasonable parameter estimate was a nominal 10 mmHg between-group difference in the change from baseline in MAP, with a group standard deviation of 13 mmHg. A minimum of 30 patients per treatment arm was estimated to be required to detect this difference, with 80% power to prevent a Type II error (which would incorrectly accept the null hypothesis that a statistical test would not demonstrate a treatment difference) and an overall two-sided significance level (alpha) of 0.05. If a formal interim analysis of the primary study endpoint was deemed appropriate, alpha levels of 0.03 and 0.02 would be assumed for the interim and final data reviews. On the basis of these parameters, between 40 and 50 patients per treatment arm were required. Approximately 200 patients were recruited during the course of the trial; about half were scheduled to have completed the trial by 1 year.

For the primary efficacy analysis, we used a last-observation-carried-forward (LOCF) approach to replace missing values in the intent-to-treat (ITT) population. For all secondary efficacy analyses, we used the observed data. For demographic and baseline characteristics and safety endpoints, we analyzed each variable using all available data. Patients with missing data were excluded only from the analyses for which data were not available.

**Figure 1:**
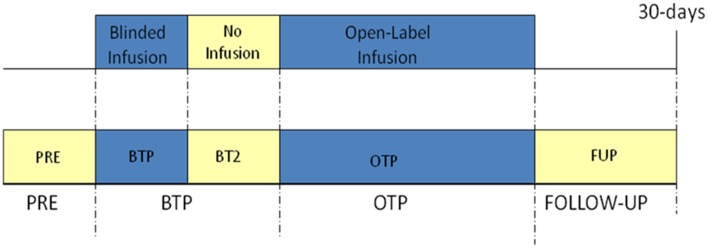
**Schematic of study flow**. PRE, pre-study drug administration period; BTP, blinded treatment phase; BT2, blinded treatment phase II; OTP, open-label treatment phase. See manuscript text for description of each phase.

The study was divided into the following four study periods (see Figure 1):

1. *Pre-study drug administration*, which encompassed up to 14 days preceding the start of the surgery or procedure. During this time, informed consent/assent, randomization, and other enrollment procedures were performed. Demographic information [gender, age, race, height (length for neonates and infants), and weight], operative information (e.g., preoperative diagnosis and name of surgical or medical procedure), and screening vital signs were collected.
2. *Blinded study drug administration*, which began with the start of study drug administration following stabilization of anesthesia. During this time, patients were administered a blinded dose of SNP for up to 30 min. To improve the safety of onset of effect, the blinded dose was initiated at one-third the full randomized rate for 5 min and was then increased to two-thirds the full rate for 5 additional minutes, after which time the dose was increased to the full rate if the target blood pressure was not attained for the final 20 min of the 30-min blinded period. The blinded dose was chosen from a pre-designed randomization schedule and included doses of 0.3, 1, 2, and 3 mcg/kg/min. Blinded study drug administration was started either before or after the surgical incision. If the blinded study drug was initiated before the incision, administration was also completed prior to incision, so the incision was not made during the 30-min blinded study drug administration period. This period ended at the start of open-label study drug infusion or at the completion of the blinded infusion, if no open-label study drug was given.
3. *Open-label study drug administration*, which is also referred to as the open-label treatment phase (OTP), began with the start of open-label study drug infusion. This phase lasted at least 90 min, during which time SNP was initiated at a dose deemed appropriate by the investigator and was gradually adjusted to reach a target MAP defined by the investigator for each individual patient, in keeping with the clinical presentation and the needs of the patient. The target MAP was determined and recorded before infusion of the study drug was initiated and could not be less than 50 mmHg (or 40 mmHg for patients younger than 30 days of age). The OTP ended at the completion of open-label infusion. In some cases, patients continued to receive SNP after the period was completed when it was clinically indicated. In these cases, SNP was considered concomitant medication and not study drug.
4. *Follow-up* was started immediately after infusion of the study drug was concluded and ended after 30 days, during which time any serious adverse events (SAEs) were monitored and recorded.

## Results

A total of 203 subjects were enrolled in the ITT group. The demographics of enrolled subjects are presented in Table 2 and are listed according to blinded dosing group.

**Table 2:** **Demographic data displayed by randomized treatment group**.

**Baseline demography summary statistic/category descriptor**	**Treatment group**				
	**0.3 μg/kg/min (*N* = 50)**	**1 μg/kg/min (*N* = 49)**	**2 μg/kg/min (*N* = 53)**	**3 μg/kg/min (*N* = 51)**	**Total (*N* = 203)**
**AGE AT SCREENING (MONTHS)**					
Mean ± *SD*	110.30 ± 75.424	114.74 ± 71.479	114.21 ± 73.864	111.30 ± 76.429	112.64 ± 73.810
Median	143.50	138.40	136.90	143.20	140.30
Min, Max	0.7, 200.8	2.1, 198.0	0.2, 200.1	0.6, 203.8	0.2, 203.8
**SEX**					
Male	14 (28.0%)	21 (42.9%)	20 (37.7%)	19 (37.3%)	74 (36.5%)
Female	36 (72.0%)	28 (57.1%)	33 (62.3%)	32 (62.7%)	129 (63.5%)
**ETHNICITY**					
Hispanic or Latino	11 (22.0%)	11 (22.4%)	12 (22.6%)	13 (25.5%)	47 (23.2%)
Not Hispanic or Latino	39 (78.0%)	38 (77.6%)	41 (77.4%)	38 (74.5%)	156 (76.8%)
Not reported	0 (0.0%)	0 (0.0%)	0 (0.0%)	0 (0.0%)	0 (0.0%)
Missing	0 (0.0%)	0 (0.0%)	0 (0.0%)	0 (0.0%)	0 (0.0%)
**RACE**					
American Indian or Alaska native	0 (0.0%)	0 (0.0%)	0 (0.0%)	0 (0.0%)	0 (0.0%)
Asian	1 (2.0%)	3 (6.1%)	4 (7.5%)	3 (5.9%)	11 (5.4%)
Black or African American	7 (14.0%)	4 (8.2%)	5 (9.4%)	4 (7.8%)	20 (9.9%)
Native Hawaiian or Other Pacific Islander	1 (2.0%)	0 (0.0%)	0 (0.0%)	0 (0.0%)	1 (0.5%)
White or Caucasian	40 (80.0%)	42 (85.7%)	42 (79.2%)	40 (78.4%)	164 (80.8%)
Other	1 (2.0%)	0 (0.0%)	0 (0.0%)	0 (0.0%)	1 (0.5%)
Reported more than one race	2 (4.0%)	2 (4.1%)	2 (3.8%)	1 (2.0%)	7 (3.4%)
Not reported	2 (4.0%)	2 (4.1%)	4 (7.5%)	6 (11.8%)	14 (6.9%)

Study participants were classified into one of the following five categories according to the surgical or medical procedure performed:

1. Spinal fusion (*n* = 106, 52.2%).
2. Craniofacial reconstruction (*n* = 71, 35.0%).
3. Coarctation of the aorta repair (*n* = 14, 7.0%).
4. Cerebral angiography (*n* = 4, 2.0%).
5. Other (*n* = 8, 3.9%).

**Figure 2:**
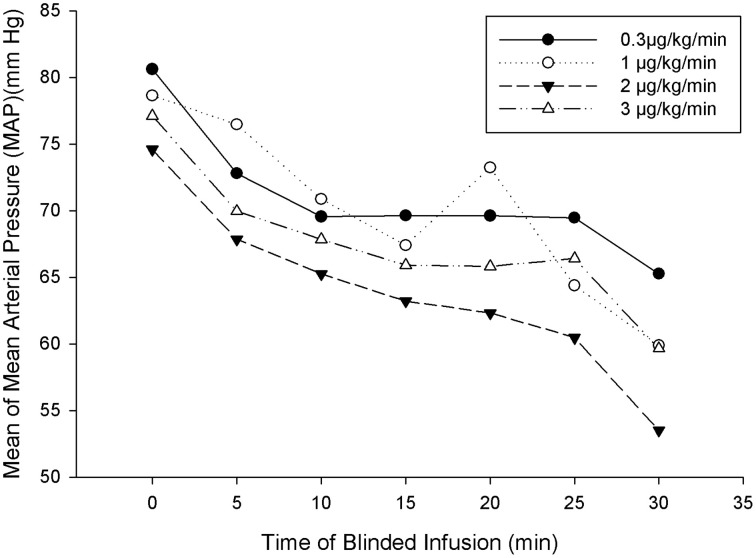
**Mean MAP over time for ITT patients for the 30-min blinded phase**.

The primary efficacy parameter was the change from baseline MAP 30 min after initiation of SNP infusion during the blinded study drug period. A comparison of change from baseline in MAP across the SNP dose groups is summarized by mean at baseline after 30 min, and change from baseline for the ITT population (Table 3). This comparison includes an analysis of within-group differences. For the ITT population, administration of SNP statistically significantly reduced MAP 30 min after the start of infusion at each of the four SNP doses (*p* < 0.001). A dose response was observed for the change in MAP from baseline 30 min after the start of infusion; larger mean reductions from baseline in MAP were observed with increasing SNP dose (0.3–2 μg/kg/min). Notably, the reductions in MAP from baseline at the 1-, 2-, and 3-μg/kg/min SNP doses (−17.0, −20.0, and −16.6 mmHg, respectively) were larger than the reduction observed at the 0.3-μg/kg/min dose (−11 mmHg). The mean MAP over time is presented in Figure 2 for the ITT population. Figure 2 graphically depicts our finding that all four SNP doses decreased MAP from baseline, with larger reductions seen with the 1-, 2-, and 3-μg/kg/min doses compared with the 0.3-μg/kg/min SNP dose. Figure 2 also demonstrates that increasing SNP dose produced greater reductions in MAP with increasing SNP dose (0.3–2 μg/kg/min). The stratified age groups all had a significant reduction in blood pressure during the blinded phase (infants *p* < 0.031, preschool *p* < 0.032, school age *p* < 0.020, and adolescent *p* < 0.026).

**Table 3:** **Primary efficacy—change from baseline in MAP (mm Hg) after 30 min double-blinded infusion**.

**Assessment endpoint category**	**Statistic**	**Treatment**				
		**0.3 μg/kg/min (*N* = 50)**	**1 μg/kg/min (*N* = 49)**	**2 μg/kg/min (*N* = 53)**	**3 μg/kg/min (*N* = 51)**	**Total (*N* = 203)**
**BASELINE OBSERVED**						
	Mean ± *SD*	76.3 ± 11.37	76.9 ± 14.50	73.5 ± 11.50	76.3 ± 12.06	75.7 ± 12.37
	Median	76.0	74.0	73.0	75.0	75.0
	Min, Max	58, 108	55, 120	45, 102	55, 102	45, 120
**30 min**						
	Mean ± *SD*	65.3 ± 13.30	59.9 ± 15.43	53.5 ± 12.09	59.6 ± 17.82	59.5 ± 15.28
	Median	65.0	58.0	49.0	56.0	58.0
	Min, Max	36, 89	40, 123	35, 92	31, 125	31, 125
**CHANGE FROM BASELINE**						
	Mean ± *SD*	−11.0 ± 15.68	−17.0 ± 12.88	−20.0 ± 15.95	−16.6 ± 18.63	−16.2 ± 16.16
	Median	−9.5	−19.0	−21.0	−19.0	−18.0
	Min, Max	−59, 24	−46, 36	−59, 24	−52, 49	−59, 49
	Within-group *P*-value	<0.001	<0.001	<0.001	<0.001	<0.001
	95% Confidence interval	−15.45, −6.55	−20.70, −13.30	−24.38, −15.58	−21.87, −11.39	−18.44, −13.97

Baseline is defined as the data collected closest in time to and just before the initial infusion of SNP.

P-value and 95% confidence interval are based on a paired t-test.

We determined the proportion of patients who achieved the target MAP in each treatment group, as well as the mean infusion rate at the target MAP. Overall, 98.5% of the patients achieved the target MAP. The majority of these patients first achieved target MAP during the blinded study drug period while they were actively receiving the infusion (72.9%). All of the patients (100%) in the 1-μg/kg/min and 3-μg/kg/min SNP dose groups achieved target MAP, and 96 and 98.1% of the patients in the 0.3-μg/kg/min and 2-μg/kg/min SNP groups achieved target MAP overall. The mean infusion rate for which the target MAP was achieved was 1.07 μg/kg/min overall.

## Safety

The mean total SNP dose was 43.01 μg/kg for neonates, 22.81 μg/kg for infants, 15.18 μg/kg for preschool patients, 18.28 μg/kg for elementary school-age patients, and 16.88 μg/kg for adolescents. Because the blinded dose was increased by graduated amounts, we analyzed the data to assess whether each age group tolerated the increasing dose and whether a group's members achieved the maximum blinded infusion rate. All five of the neonates (100.0%) received the 1/3, 2/3, and full SNP dose. All 50 infants (100.0%) received the one-third SNP dose, 92.0% received the two-thirds SNP dose, and 72.0% received the full SNP dose. All 12 preschool patients (100.0%) received the one-third SNP dose and two-third SNP dose, and 58.3% received the full SNP dose. All 43 school-age patients (100.0%) received the one-third SNP dose, 83.7% received the two-thirds SNP dose, and 51.2% received the full SNP dose. All 93 adolescents (100.0%) received the one-third SNP dose, 75.3% received the two-thirds SNP dose, and 45.2% received the full SNP dose.

Adverse events were coded using the MedDRA (version 8.1). Treatment-emergent adverse events (TEAEs) were defined as follows: an AE first observed after the initiation of the study drug (blinded or open-label) or a representation of an exacerbation (usually in severity) of a pre-existing condition observed prior to treatment. An overall summary of tolerability and AEs is provided in Table 4. All 203 patients (100.0%) experienced one or more TEAEs. Eighteen patients (8.9%) experienced one or more treatment-emergent SAEs, and 166 patients (81.8%) experienced treatment-related TEAEs (probably or possibly related). These TEAEs were classified as severe in 97 patients (47.8%), moderate in 73 patients (36.0%), and mild in 33 patients (16.3%). Sixty-six patients (32.5%) had at least one TEAE severe enough to lead to study drug withdrawal, but no patients experienced TEAEs severe enough to discontinue the study. There were no deaths during the study.

**Table 4:** **Overall summary of tolerability/adverse events for ITT safety population**.

**Patients included**	**Treatment group**				
**Measure of toleration category**	**0.3 μg/kg/min**	**1 μg/kg/min**	**2 μg/kg/min**	**3 μg/kg/min**	**Total**
**OVERALL**					
Number of patients	50	49	53	51	203
*N* (%) of Patients with at least one TEAE	50 (100.0%)	49 (100.0%)	53 (100.0%)	51 (100.0%)	203 (100.0%)
*N* (%) of Patients with at least one TESAE	5 (10.0%)	4 (8.2%)	5 (9.4%)	4 (7.8%)	18 (8.9%)
*N* (%) of Patients with TEAE leading to study drug withdrawal	19 (38.0%)	18 (36.7%)	13 (24.5%)	16 (31.4%)	66 (32.5%)
***N* (%) OF PATIENTS WITH TEAE BY SEVERITY**					
Mild	8 (16.0%)	10 (20.4%)	7 (13.2%)	8 (15.7%)	33 (16.3%)
Moderate AE	19 (38.0%)	19 (38.8%)	20 (37.7%)	15 (29.4%)	73 (36.0%)
Severe AE	23 (46.0%)	20 (40.8%)	26 (49.1%)	28 (54.9%)	97 (47.8%)
***N* (%) OF PATIENTS WITH TEAE BY RELATIONSHIP TO STUDY DRUG**					
Probably related	29 (58.0%)	32 (65.3%)	40 (75.5%)	36 (70.6%)	137 (67.5%)
Possibly related	12 (24.0%)	6 (12.2%)	2 (3.8%)	9 (17.6%)	29 (14.3%)
Probably not related	1 (2.0%)	5 (10.2%)	0 (0.0%)	3 (5.9%)	9 (4.4%)
Not related	8 (16.0%)	6 (12.2%)	11 (20.8%)	3 (5.9%)	28 (13.8%)
*N* (%) of deaths	0 (0.0%)	0 (0.0%)	0 (0.0%)	0 (0.0%)	0 (0.0%)

TEAE, treatment-emergent adverse event; TESAE, treatment-emergent serious adverse event.

At each level of summarization, patients reporting more than one AE were counted only once under the strongest relationship and/or severity.

Neonates, birth to < 30 days; infant, 30 days to < 2 years; preschool, 2 years to < 6 years; school age, 6 years to < Tanner stage III; adolescent, Tanner stage III to < 17 years.

The most frequently reported TEAEs (>20%) occurred in the following system organ classes (SOCs): blood and lymphatic system disorders; gastrointestinal disorders; general disorders and administration site conditions; injury, poisoning, and procedural complications; and vascular disorders. The most frequently reported TEAEs (>20%) were hypotension (75.4%), pyrexia (35.0%), operative hemorrhage (33.0%), vomiting (31.5%), nausea (30.5%), hypertension (25.6%), anemia (24.1%), and rebound hypertension (23.6%). No dose-response relationship was apparent for TEAEs, because all patients in each of the four SNP dose groups experienced at least one TEAE (Pyrexia occurring within 24 h of surgery was considered an AE only if it was severe, because it is a condition associated with surgical procedures).

We performed co-oximetry on arterial blood gas samples and measured serum methemoglobin, lactate concentrations, central venous oxygen saturation (in patients with an indwelling central venous catheter), whole blood cyanide concentrations, and plasma and urine thiocyanate concentrations. We also assessed acid-base status. No cyanide toxicity was reported and no measureable cyanide levels were detected.

## Discussion

We performed a randomized, double-blind, parallel-group, dose-ranging, effect-controlled, multicenter study to examine the effects of SNP in pediatric patients requiring controlled hypotension during a surgical or medical procedure. The goals of the study were to prove the efficacy of SNP in various pediatric age groups and to assess the safety of SNP administration in pediatric patients requiring controlled reduction of blood pressure. In addition, analysis of the data was designed to establish the starting and maximum infusion rates that afford optimum blood pressure control and a safe dosing regimen in pediatric patients.

The following results describe the relationship between SNP dose and changes in blood pressure in pediatric patients:

1. Administration of SNP resulted in statistically significant reductions in MAP from baseline 30 min after the initial start of blinded study drug infusion for each of the SNP doses (0.3, 1, 2, and 3 μg/kg/min).
2. A dose response for changes in MAP from baseline determined within 30 min of blinded study drug infusion was observed in the ITT population as a general trend, suggesting larger mean reductions with increasing SNP doses (0.3–2 μg/kg/min). However, the dose response should not be considered linear because the SNP dose increases were not equidistant. The maximum effect on change in MAP from baseline at 30 min after the infusion started was generally observed at the 2-μg/kg/min SNP dose. The effect of the 3-μg/kg/min SNP dose on MAP was less than that observed with the 1- and 2-μg/kg/min dose. This was an unexpected finding that could be due to the protocol design, which may have selected for non-responders, as evidenced by the number of ITT patients in the 2- and 3-μg/kg/min SNP dose groups (13, 23.6%; and 18, 35.3%) compared with those in the 0.3- and 1-μg/kg/min SNP dose groups (38, 74.5%; and 23, 46.9%).
3. Overall, 98.5% of the patients achieved target MAP during therapy. The majority of patients (72.9%) first achieved target MAP during the blinded study drug period.

Our study's safety results included the following: 18 patients (8.9%) experienced one or more treatment-emergent SAEs. Vascular disorders were the most common study-drug-related TEAEs (>5% overall) and comprised the most common severe, specific drug-related TEAEs. Hypotension was the AE that led to the most study drug withdrawals. No patients experienced TEAEs leading to study discontinuation and no deaths occurred during the study.

An enormous safety concern is cyanide poisoning, but it can be addressed. One way the development of poisoning happens is as follows: The metabolism of SNP in red blood cells (RBCs) results in the production of 5 molecules of cyanide, one of which is then converted to thiocyanate (SCN) or is bound to methemoglobin to form cyanomethemoglobin. This conversion occurs enzymatically via two sulfur transferase systems: (1) rhodanase (the primary pathway) and (2) beta-mercaptopyruvate-cyanide sulfurtransferase (the secondary pathway). Rhodanase is ubiquitous in the body but highly concentrated in the liver. Rhodanase catalyzes the transfer of sulfur from a donor molecule such as sodium thiosulfate (NaS_2_O_3_) to cyanide, to form SCN, which is subsequently eliminated in the urine, where it serves as a marker of cyanide exposure. However, this conversion is limited by the availability of sulfur donor molecules. A life-saving intervention in the event of possible acute cyanide intoxication is to provide the patient exogenous sulfur donors such as Na_2_S_2_O_3_ (Pasch et al., 1983; Cole and Vesey, 1987).

Despite the widespread use of SNP, there is a paucity of information on its safety, efficacy, and pharmacokinetic/pharmacodynamic (PK/PD) relationships in children. Davies et al. and Bennett and Abbott described their retrospective experience with SNP used to induce deliberate hypotension in small cohorts of children (Davies et al., 1975; Bennett and Abbott, 1977). Both observed that younger patients required more SNP than older ones to achieve comparable degrees of blood pressure control. In their small retrospective cohort, Bennett and Abbott recommended that doses of 10 μg/kg/min were necessary to achieve satisfactory blood pressure response. Davies et al described three possible responses to SNP administration in children: (1) a constant response to “conventional” doses < 3 mg/kg, (2) a tachyphylactic response characterized by continuously escalating dose requirement (>3 mg/kg) to achieve a satisfactory blood pressure, and (3) resistance to the blood pressure lowering effects of the drug (Davies et al., 1975). Yaster et al. compared SNP and nitroglycerine for inducing hypotension in a group of 14 adolescents (Yaster et al., 1986). They found that doses of SNP between 6 and 8 μg/kg/min were superior to doses of nitroglycerine at any dose for reliably inducing hypotension for children and adolescents undergoing scoliosis, craniofacial, or hepatic surgery.

Hersey et al. performed a randomized trial comparing SNP to the dihydropyridine calcium-channel antagonist nicardipine in 20 healthy adolescents with idiopathic scoliosis undergoing spinal fusion (Hersey et al., 1997), and they easily obtained target blood pressures in both groups and operating conditions were comparable. They found that time-to-restoration of baseline blood pressure after termination of the infusion was significantly longer in the nicardipine group than in the SNP group, but, interestingly, blood loss was significantly greater in the SNP group. Details on SNP dose requirements were not provided. Przybylo et al. described cyanide and SCN blood levels in 10 children who received SNP at doses up to 10 μg/kg/min (mean infusion rate 6 μg/kg/min) while undergoing cardiopulmonary bypass for repair of complex congenital cardiac defects (Przybylo et al., 1995). Cyanide levels rose as a function of time while SNP was infused and rapidly fell when SNP was discontinued. Despite the fact that some children demonstrated plasma cyanide levels above the generally accepted threshold of 0.5 μg/mL, no patients developed clinically apparent toxicity. Kazim et al. questioned the validity of the results of Przybylo et al's study because of the cyanide assay methods used (Kazim et al., 1996). Linakis et al. retrospectively examined physician-ordering practices for blood cyanide levels in children receiving SNP (Linakis et al., 1991). They sought to determine how the laboratory determinations were used to monitor patients and if there was clinically apparent toxicity in children found to have cyanide concentrations exceeding the “normal” limit of 0.5 μg/mL. They found poor correlation between blood cyanide concentration and dose or duration of therapy in patients whose cyanide levels were “toxic.” Thiocyanate determinations were normal and no child manifested signs or symptoms of cyanide toxicity. They concluded that further pediatric studies were needed.

On the basis of this study's efficacy and safety data, we make several recommendations:

1. A reasonable starting dose of SNP in pediatric patients requiring deliberate induced hypotension is 0.3 μg/kg/min; in our study the mean infusion rate was approximately 1 μg/kg/min.
2. Clinicians should increase the infusion rate to achieve the desired reduction in blood pressure.
3. Because our data demonstrate wide variability in patient responses to SNP, especially in regard to baseline MAP, basal metabolic rate, and age, clinicians should use their experience and the patient's clinical situation as a basis for determining both the starting and maintenance infusion rates.
4. Given that SNP is titrated to MAP response in most situations, SNP dosing is already implicitly individualized. However, modeling hemodynamic responses suggests ways to more rapidly achieve target MAP endpoints and also suggests that an algorithm-guided approach as opposed to the currently employed empirical dose-titration approach may be desirable.

In summary, individual patient dosing guidance must reflect both the target and the population.

## The SNP investigators group

Peter Davis, MD (Children's Hospital of Pittsburg), Shoba Malviya, MD (Mott Hospital, Ann Arbor, MI), Lynne Maxwell, MD (Children's Hospital of Philadelphia), David McLeod, MD (Duke University Medical Center), Gary Scott, MD (Children's Hospital Los Angeles), David A. Rosen, MD (West Virginia University), Steven Stayer, MD (Texas Children's Hospital), Luis Zabala, MD (Arkansas Children's Hospital).

## Funding

NO1-HD-4-3385 (NICHD, Rockville, Maryland).

### Conflict of interest statement

The Reviewer Daniel J. Licht declares that, despite being affiliated to the same institution as the authors Jeffrey Barrett and Lynne Maxwell, the review process was handled objectively and no conflict of interest exists. The authors declare that the research was conducted in the absence of any commercial or financial relationships that could be construed as a potential conflict of interest.
